# The Neural Origin of Nociceptive-Induced Gamma-Band Oscillations

**DOI:** 10.1523/JNEUROSCI.0255-20.2020

**Published:** 2020-04-22

**Authors:** Lupeng Yue, G.D. Iannetti, Li Hu

**Affiliations:** ^1^CAS Key Laboratory of Mental Health, Institute of Psychology, Chinese Academy of Sciences, Beijing 100101, People's Republic of China; ^2^Department of Psychology, University of Chinese Academy of Sciences, Beijing 100049, People's Republic of China; ^3^Neuroscience and Behaviour Laboratory, Istituto Italiano di Tecnologia, Rome, 00161, Italy; ^4^Department of Neuroscience, Physiology and Pharmacology, University College London, London WC1E 6BT, United Kingdom

**Keywords:** biomarkers, gamma-band oscillations, interneurons, pain, primary motor cortex, primary somatosensory cortex

## Abstract

Gamma-band oscillations (GBOs) elicited by transient nociceptive stimuli are one of the most promising biomarkers of pain across species. Still, whether these GBOs reflect stimulus encoding in the primary somatosensory cortex (S1) or nocifensive behavior in the primary motor cortex (M1) is debated. Here we recorded neural activity simultaneously from the brain surface as well as at different depths of the bilateral S1/M1 in freely-moving male rats receiving nociceptive stimulation. GBOs measured from superficial layers of S1 contralateral to the stimulated paw not only had the largest magnitude, but also showed the strongest temporal and phase coupling with epidural GBOs. Also, spiking of superficial S1 interneurons had the strongest phase coherence with epidural GBOs. These results provide the first direct demonstration that scalp GBOs, one of the most promising pain biomarkers, reflect neural activity strongly coupled with the fast spiking of interneurons in the superficial layers of the S1 contralateral to the stimulated side.

**SIGNIFICANCE STATEMENT** Nociceptive-induced gamma-band oscillations (GBOs) measured at population level are one of the most promising biomarkers of pain perception. Our results provide the direct demonstration that these GBOs reflect neural activity coupled with the spike firing of interneurons in the superficial layers of the primary somatosensory cortex (S1) contralateral to the side of nociceptive stimulation. These results address the ongoing debate about whether nociceptive-induced GBOs recorded with scalp EEG or epidurally reflect stimulus encoding in the S1 or nocifensive behavior in the primary motor cortex (M1), and will therefore influence how experiments in pain neuroscience will be designed and interpreted.

## Abstract (precis)

Gamma-band oscillations (GBOs) elicited by transient nociceptive stimuli are one of the most promising biomarkers of pain across species. Still, whether these GBOs reflect stimulus encoding in the primary somatosensory cortex (S1) or nocifensive behavior in the primary motor cortex (M1) is debated. Here we recorded neural activity simultaneously from the brain surface as well as at different depths of the bilateral S1/M1 in freely-moving male rats receiving nociceptive stimulation.

## Introduction

There is a painstaking effort worldwide to identify objective markers of pain experience, given the inherent biases that confound subjective reports (Davis et al., 2017; Mouraux and Iannetti, 2018). Since pain perception emerges from a state of electrical activity within the nervous system, researchers have focused their efforts toward identifying features of brain activity that reflect pain objectively (i.e., brain-based pain biomarkers; Huang et al., 2013; Wager et al., 2013; Kumbhare et al., 2017). Some of these biomarkers have yielded spectacular results: they have allowed predicting the perceived intensity of transient pain (Huang et al., 2013; Wager et al., 2013), pain sensitivity across individuals (Hu and Iannetti, 2019), as well as the effectiveness of individual therapeutic effect of placebo analgesia (Tétreault et al., 2016) and of pain-relieving drugs (Woolf and Max, 2001). Still, most pain biomarkers are neither selective nor generalizable (Hu and Iannetti, 2016), because the majority of the brain responses elicited by stimuli perceived as painful are not specific (Iannetti and Mouraux, 2010; Mouraux and Iannetti, 2018). Indeed, most brain responses observed when pain is present can also be observed when pain is absent (e.g., following the presentation of salient auditory, visual, and non-nociceptive somatosensory stimuli; Mouraux and Iannetti, 2009; Mouraux et al., 2011; Liberati et al., 2016), or even in patients with congenital pain insensitivity (Salomons et al., 2016). Therefore, researchers are trying to identify neural markers of pain that are selective and generalizable.

Cortical oscillations in the gamma frequency [gamma-band oscillations (GBOs)] are currently one of the most promising selective markers of pain experience (Hu and Iannetti, 2019). Not only is the relationship between GBOs and pain independent of stimulus saliency—a factor explaining the correlation of most nociceptive-evoked responses with pain intensity (Zhang et al., 2012)—but they also reliably predict pain sensitivity across different individuals, in both humans (Hu and Iannetti, 2019) and rodents (Peng et al., 2018). Furthermore, GBO magnitude tracks the time-varying fluctuations of the intensity of clinical pain in patients, although these GBOs have been suggested to originate from the prefrontal cortex and cerebellum, and not from the primary sensorimotor cortex (Zhou et al., 2018; May et al., 2019). Functionally, there is strong evidence that GBOs are important for communications within a large network of cortical and subcortical structures (Saleem et al., 2017; Tan et al., 2019), and for this reason they have been suggested to subserve an integrating role in the generation of the conscious experience of pain (Schulz et al., 2011) and a filtering mechanism to select behaviorally relevant information for action (Gross et al., 2007).

Despite their promising role as a pain biomarker, the neural origin of pain-related GBOs remains debated (Ploner and Gross, 2019). GBOs detected in human EEG studies are typically strongest at two discrete clusters of electrodes, each above one primary sensorimotor cortex. GBOs measured contralaterally to the stimulated hand causally determine those measured ipsilaterally, probably through transcallosal transmission (Zhang et al., 2012). In direct recording from the cortical surface in rats [electrocorticography (ECoG)], GBOs are strongest at central electrodes, with a maximum contralateral to the stimulated paw, which also suggests a generator in the contralateral primary sensorimotor cortex (Peng et al., 2018). Another human study, however, suggests that GBOs largely originate from the primary motor cortex and are consequent to the transfer of information from the somatosensory to the motor areas (Schulz et al., 2012). Given the inconsistency of these results and the intrinsic inaccuracy of the source analysis of electrocortical surface data, to pin down the neural origin of GBOs sampled from the cortical surface a simultaneous recording of intracortical neural activity is imperative.

**Figure 1.**
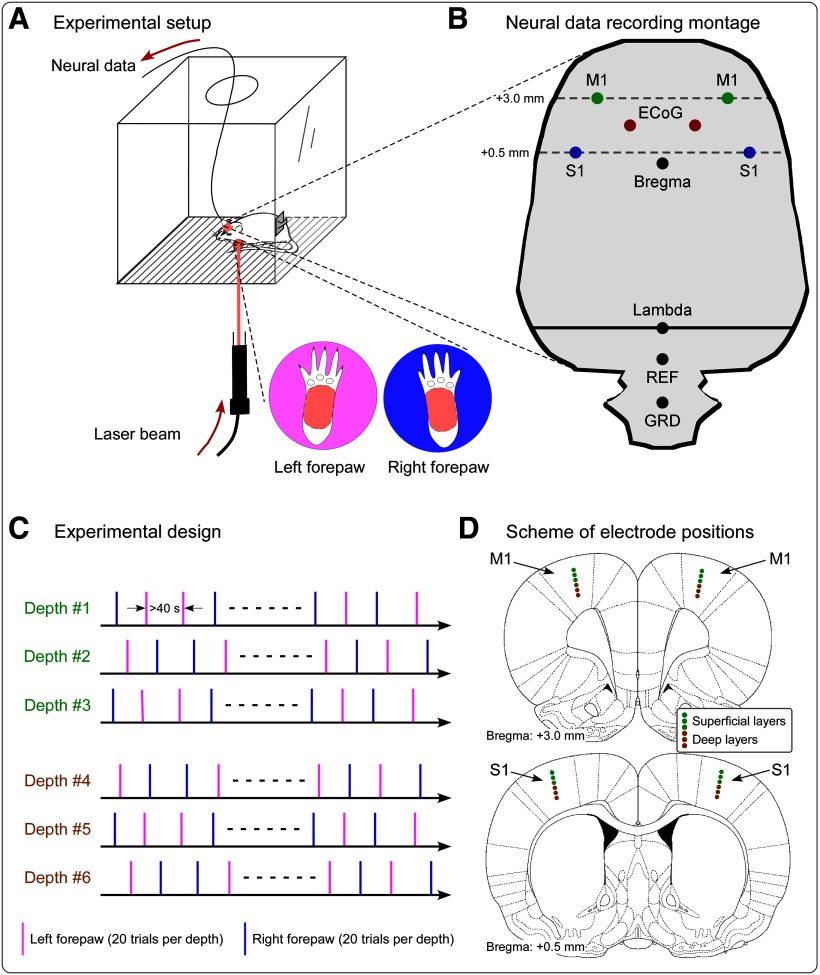
Experimental design and recording setup. ***A***, During the recording sessions, rats were free to move within a plastic chamber (30 × 30 × 30 cm^3^). When the animal was spontaneously still, laser stimuli were delivered on the plantar surface of either the left or the right forepaw through gaps on the floor of the chamber. ***C***, In each recording session, the neural activity was measured at one of six cortical depths. In each session, we delivered 20 laser pulses to the right forepaw and 20 laser pulses to the left forepaw, in pseudorandom order. The interval between two consecutive stimuli was never <40 s. ***B***, ***D***, Scheme showing the position of electrodes for the simultaneous intracortical and epidural recording. ***B***, Positioning of the four microelectrodes for the recording of LFPs and single-unit activity in the S1 and M1 contralateral and ipsilateral to the stimulation site, as well as of epidural electrodes. Intracortical microelectrodes were placed according to stereotaxic coordinates in the following positions [expressed in respect to the bregma (in mm); positive *x*-axis and *y*-axis values indicate right and anterior locations, respectively]: left S1: *x* = −4, *y* = 0.5; right S1: *x* = 4, *y* = 0.5; left M1: *x* = −3, *y* = 3; right M1: *x* = 3, *y* = 3. Epidural electrodes were placed in the following positions: left ECoG: *x* = −1.5, *y* = 1.75; right ECoG: *x* = 1.5, *y* = 1.75. Reference (REF) and ground (GRD) electrodes were placed 2 and 4 mm caudally to the lambda, on the midline. ***D***, Intracortical neural data were recorded at six different depths. Electrode positions measuring data from superficial and deep cortical layers are marked in light green and brown, respectively.

Here, we addressed this issue by simultaneously recording (1) the neural activity of populations of neurons using epidural ECoG, and (2) the local field potential (LFP) and spiking activity of single neurons in bilateral primary somatosensory cortex (S1) and primary motor cortex (M1) using intracortical microelectrodes (Fig. 1). We show that GBOs measured epidurally are strongly related to several features of the activity of neurons located in the S1 contralateral to the stimulated paw.

## Materials and Methods

### Subjects

Eight adult male Sprague Dawley rats (weighing between 400 and 450 g) were used in the experiment. Rats were housed individually in a constant temperature of 23°C under a 12 h light/dark cycle with food and water available *ad libitum*. All surgical and experimental procedures adhered to the guidelines for animal experimentation and were approved by the animal care and use committee of Peking University.

### Surgical procedures and electrode implantation

Surgical procedures are detailed in our previous publications (Hu et al., 2015; Yue et al., 2019). An array composed of four microelectrodes with a contact at the tip of the filament and two stainless steel screws, assembled in a 3D-printed module, were used to simultaneously record electrophysiological activities intracortically and epidurally (Fig. 1*B*; Yue et al., 2019). The depth of each microelectrode was adjusted using a microdrive. Each microelectrode contained five tungsten-coated filaments to increase the probability of detecting multiunit spikes (diameter, 50 μm; impedance, 300–400 kΩ; California Fine Wire Company). Stainless steel screws (diameter, 1 mm) were used as epidural electrodes. The screws were implanted into holes on the skull, without penetrating the underlying dura mater. According to coordinates given by the Paxinos and Watson (2007) atlas, the four microwire arrays were implanted in the forepaw regions of the bilateral primary somatosensory cortices [S1: anteroposterior (AP), 0.5 mm; mediolateral (ML), ±4.0 mm] and of the bilateral primary motor cortices (M1: AP, 3.0 mm; ML, ±3.0 mm; Paxinos and Watson, 2007; Moxon et al., 2008; Alloway et al., 2010). In each hemisphere, one epidural electrode was placed in between the S1 and M1 microelectrodes (AP, 1.5 mm; ML, ±1.5 mm). The reference and ground for both intracortical and epidural electrodes were on the midline, 2.0 mm and 4.0 mm caudally to the lambda, respectively. To prevent postsurgical infections, rats were injected with penicillin (60,000 U, i.p.) immediately after the surgery. Following the surgery, rats were kept in individual cages for at least 7 d before data collection. At the end of the experiment, all animals were deeply anesthetized, and the recording sites were marked by passing a direct current (20 μA for 10 s) through the microwire arrays. Rats were finally perfused, and their brains were serially sectioned to verify histologically the electrode positions determined by the lesion created by the microstimulation (Tutunculer et al., 2006).

### Nociceptive stimuli

Radiant-heat nociceptive stimuli were generated by an infrared neodymium yttrium aluminum perovskite (Nd:YAP) laser with a wavelength of 1.34 μm (Electronical Engineering), which activates directly cutaneous nociceptive terminals in the most superficial skin layers (Iannetti et al., 2006; Leandri et al., 2006; Sikandar et al., 2013). The laser beam was transmitted via an optic fiber, and its diameter was set at ∼5 mm (∼20 mm^2^) by focusing lenses. An He-Ne laser pointed to the area to be stimulated, and laser pulses were delivered on the glabrous skin of the left and right forepaws (Fig. 1*A*). Stimulus energy was 4.0 J, and pulse duration was 4 ms. The interval between two consecutive stimuli was never <40 s. To avoid nociceptor fatigue or sensitization, the target of the laser beam was displaced after each stimulus (Hu et al., 2015).

### Experimental design

Electrophysiological signals were recorded at a sampling rate of 20,000 Hz (RHD2000, Intan Tech). To sample neural activity at different cortical depths, we adjusted the depth of the implanted microelectrodes every 3 d. The initial depth of each microelectrode was set at 0.3 to ∼0.5 mm beneath the brain surface and was lowered by 200 μm at the end of each recording session. For each animal, data were collected from five to seven depths (one depth per recording session). After all recording sessions, the depth of the microelectrodes was between 1.3 and 1.7 mm from the brain surface (Fig. 1*D*).

Before each recording session, rats were placed for 1 h into a plastic chamber (30 × 30 × 30 cm^3^) to be familiarized with the recording environment. During the recording sessions, rats could move freely in the chamber. Laser stimuli were delivered on the plantar surface of left and right forepaws through gaps on the floor of the chamber when the animal was spontaneously still. Twenty laser pulses were delivered to each stimulation site (left or right forepaw), for a total of 40 pulses (Fig. 1*C*). White noise (70 dB SPL) was played throughout the recording session; this procedure is important as it avoids the activation of the auditory system by the laser-generated ultrasounds, and thereby allows for a selective recording of brain responses related to the activation of the nociceptive system (Hu et al., 2015). Rats were video recorded throughout the experiment to identify the occurrence of a withdrawal elicited by laser stimuli. Rats showed an obvious withdrawal in ∼80% of the trials and no obvious withdrawal in ∼20% of the trials. Trials without withdrawal were considered as no-pain trials and were discarded from the following analyses.

### Data analysis

#### Time–domain analysis

Electrophysiological data were preprocessed using NDManager (Hazan et al., 2006), and were analyzed using EEGLAB (Delorme and Makeig, 2004) and in-house MATLAB functions. To extract stimulus-evoked responses from LFPs and ECoG signals, electrophysiological data were downsampled to 1250 Hz, bandpass filtered between 1 and 100 Hz, and notch filtered between 48 and 52 Hz. Peristimulus epochs were extracted from the continuous data using a time window of 1500 ms (−500 to +1000 ms with respect to stimulus onset), and were baseline corrected using the prestimulus interval. Sessions in which the data were contaminated by gross artifacts in >50% of trials or collected from damaged electrodes were discarded. As a result, electrophysiological data from 31 sessions were included in the analysis, as follows: 16 sessions in which the microelectrode sampled activity from superficial layers (<0.9 mm from cortical surface, mainly layers II–IV) and 15 sessions in which the microelectrode-sampled activity from deep layers (>1.1 mm from cortical surface, mainly layers V/VI). The rationale for looking separately at data sampled from superficial and deep layers was the fact that gamma-band oscillations are more prominent in superficial layers, where local recurrent connections are more abundant than in deep layers (Allitt et al., 2017; Bruyns-Haylett et al., 2017).

For each subject, session, and experimental condition, single-trial LFP waveforms in the time domain were averaged. Peak latency and amplitude of the N1 wave were measured from each single-session average waveform. The N1 wave was defined as the most negative deflection between 100 and 200 ms after the onset of the nociceptive laser stimulus (Xia et al., 2016). Single-session average waveforms were subsequently averaged to obtain the group-level LFP waveforms.

#### Time–frequency analysis

Time–frequency distributions (TFDs) of single-trial brain responses were calculated using a windowed Fourier transform with a fixed 200 ms Hanning window. A complex time–frequency spectrum, *F*(*t, f*), was estimated for each trial, from −500 to 1000 ms (in steps of 2 ms) in latency and from 1 to 100 Hz (in steps of 1 Hz) in frequency. The resulting spectrogram, $P(t,\;f)\;=\;|F(t,\;f){|}^{\text{2}}$, represents the power spectral density as a joint function of time and frequency at each time–frequency point. Single-trial spectrograms were first averaged across trials and then baseline corrected by dividing the baseline-subtracted power of each frequency by the average power within the prestimulus interval of that same frequency (−400 to −100 ms relative to stimulus onset; Hu et al., 2014). To provide a detailed characterization of gamma-band oscillations elicited by nociceptive laser stimuli, we first calculated the time course of GBO magnitude by averaging TFDs across gamma frequencies (i.e., 70–100 Hz). We subsequently performed a point-by-point statistical analysis to identify time intervals in which the GBO magnitude was different between recording sites. Specifically, for each time point, we performed a two-way repeated-measures ANOVA to assess the possible effects of 'hemisphere' (two levels: contralateral and ipsilateral to stimulation side), 'brain region' (two levels: S1 and M1), and their interaction. To account for multiple comparisons across time, the significance level (expressed as *p* value) was corrected using a false discovery rate (FDR) procedure. To control for false-positive observations, only intervals with a *p* value smaller than a defined threshold (*p*_FDR_ < 0.05) for >20 ms were considered in the subsequent quantitative analysis. To provide a better visualization of the modulation of GBO magnitude, we extracted the summary value of GBO magnitude from each significant time interval by computing the mean of all time points within the interval, for each subject, session, and experimental condition.

#### Cross-correlation of epidural and intracortical GBOs

To estimate the temporal relationship between the GBOs recorded intracortically and epidurally, we calculated the cross-correlation of GBO instantaneous amplitudes elicited by nociceptive laser stimuli (Adhikari et al., 2010). Specifically, single-trial GBO responses, recorded both intracortically and epidurally, were bandpass filtered between 70 and 100 Hz in the time interval between 100 and 250 ms after stimulus onset. The instantaneous amplitude of single-trial GBOs was estimated using the Hilbert transform. The GBO instantaneous amplitude measured intracortically (i.e., in contralateral and ipsilateral S1 and M1) was cross-correlated with the GBO instantaneous amplitude measured epidurally (i.e., using the average of the two ECoG recording sites), with the lag of one signal over the other ranging from −100 to 100 ms. The time lag at which cross-correlation coefficients peaked was considered to represent the precedence relationship between the two signals. The distribution of the time lags of cross-correlation peaks was obtained for each recording site and experimental condition.

#### Phase consistency between epidural and intracortical GBOs

To test the similarity between the phase of GBOs sampled intracortically and epidurally, we estimated their phase consistency using the debiased weighted phase lag index (WPLI) implemented in FieldTrip (Oostenveld et al., 2011). WPLI is a measure of phase synchronization widely adopted in EEG/ECoG connectivity studies (Lau et al., 2012; Ortiz et al., 2012; Hardmeier et al., 2014). The debiased version of WPLI (Vinck et al., 2011) has been demonstrated to be robust to volume conduction effects: in other words, debiased WPLI does not overestimate the phase synchronization due to volume conduction effects of uncorrelated noise sources. In addition, compared with other connectivity measures (e.g., phase lag index), WPLI is less sensitive to noise, thus providing more reliable information about the true phase consistency (Vinck et al., 2011). We estimated debiased WPLI values between GBOs sampled intracortically and epidurally for each recording site and experimental condition.

#### Spike detection

To measure the spiking activity of single units, electrophysiological data collected from microelectrodes were first high-pass filtered at 200 Hz. Time intervals in which the amplitude variance exceeded 2 SDs were considered to contain multiunit spikes. Time intervals containing multiunit spiking activity were subsequently decomposed using a principal component analysis, and single-unit activity (i.e., individual spikes) was sorted automatically using KlustaKwik, followed by manual adjustment using the software Klusters (Hazan et al., 2006). Spikes were classified as being generated from putative interneurons or pyramidal neurons on the basis of the duration of the action potential, defined as the latency difference between the trough and the peak of the waveform (Mitchell et al., 2007; Yokoi and Komatsu, 2010; Woloszyn and Sheinberg, 2012; Jacob et al., 2016). Based on the bimodal distribution of the durations of all recorded spikes, a data-driven approach was adopted to determine the threshold to separate interneurons from pyramidal neurons (i.e., the pit between the two peaks of the distribution; Mitchell et al., 2007; Woloszyn and Sheinberg, 2012). To minimize the risk of misclassifying units, spikes whose duration was ±50 μs from the threshold were discarded. Specifically, for signals collected from bilateral S1, units with spike durations <400 μs were classified as interneurons, and units with spike durations >500 μs were classified as pyramidal neurons. For signals collected from bilateral M1, putative interneurons and pyramidal neurons had spike durations <450 and >550 μs, respectively. Units without spikes within the 1000 ms after stimulus onset were excluded from further analyses.

To estimate the modulation of spike firing by laser stimuli and thus calculate spike density functions, we segmented the data using a window analysis time of 1500 ms (−500 to +1000 ms with respect to stimulus onset). For each trial, the spike-firing rate was binned using a 100 ms window and normalized to the baseline using a *z*-score (i.e., by dividing the baseline-subtracted value by the SD within the prestimulus interval). Spikes with a firing rate <1 Hz within the 1000 ms after the stimulus onset were excluded for further analyses.

To identify neurons whose firing rate was modulated by the occurrence of laser stimuli, we compared the mean firing rate within the 500 ms after stimulus onset with the mean firing rate within the 500 ms before stimulus onset, using a paired-sample *t* test. On the basis of the result of this test, units were classified as having an excitatory response (i.e., strong evidence of a higher firing rate in the poststimulus interval than in the prestimulus interval), no response (i.e., no evidence of a different firing rate in the prestimulus and poststimulus intervals), or an inhibitory response (i.e., strong evidence of a lower firing rate in the poststimulus interval than in the prestimulus interval). The proportion of neurons showing different types of responses was calculated at each of the four recording sites (i.e., bilateral S1 and M1).

#### Spike-field coherence

To assess the relationship between spiking activity sampled at each of the four intracortical recording sites and gamma-band oscillations sampled epidurally with ECoG, we calculate the spike-field coherence (SFC) using the Chronux toolbox (Bokil et al., 2010). Specifically, in this analysis the Fourier transforms of the two signals (*x*, spike firing; *y*, gamma-band oscillations) are used to calculate multitaper estimates for the spectrum *S_x_*(*f*), for the spectrum *S_y_*(*f*), and the multitaper estimate across spectra *S_xy_*(*f*) (Womelsdorf et al., 2006). Absolute coherence was estimated as follows: $C_{xy}(f)\;=\;S_{xy}(f)/{\left[S_{x}(f)\;*\;S_{y}(f)\right]}^{\text{1/2}}$. The coherence value, which is calculated separately for each frequency, is 1 if the phase and amplitude of the two signals covary constantly, and 0 if there is no phase and amplitude relationship between the two signals (Jarvis and Mitra, 2001; Womelsdorf et al., 2006). Time–frequency distributions of spike-field coherence at gamma frequencies were calculated using a 250 ms sliding window, with a step of 100 ms. For each trial and frequency, spike-field coherence was expressed as a *z*-score by first subtracting the mean of the prestimulus interval, and then dividing by the SD of the prestimulus interval.

#### Statistical analyses

Two-way repeated-measures ANOVAs were performed to assess the effect of the experimental factors hemisphere (two levels: contralateral and ipsilateral to stimulation side) and brain region (two levels: S1 and M1) on a number of laser-evoked responses measured with intracortical electrodes, as follows: (1) LFP responses at superficial and deep layers (i.e., N1 latency and amplitude, GBO magnitude); (2) WPLI values; (3) spike-firing rates; and (4) coherence between spikes and epidural gamma-band oscillations (i.e., SFC). When ANOVA suggested the presence of interaction effects, *post hoc t* tests with Bonferroni correction were performed.

Wilcoxon rank sum tests were performed to assess whether the time lag distribution of the cross-correlation between GBOs sampled intracortically and epidurally was different from zero.

Fisher's exact tests were performed to compare the proportion of neurons showing different laser-evoked modulation of spiking (i.e., increase, no change, decrease) across the four recording sites. The data were presented as the mean ± SEM, and the degree of evidence was estimated on the basis of *p* values (Colquhoun, 2014).

## Results

### Laser-evoked field potentials in the time domain

**Figure 2.**
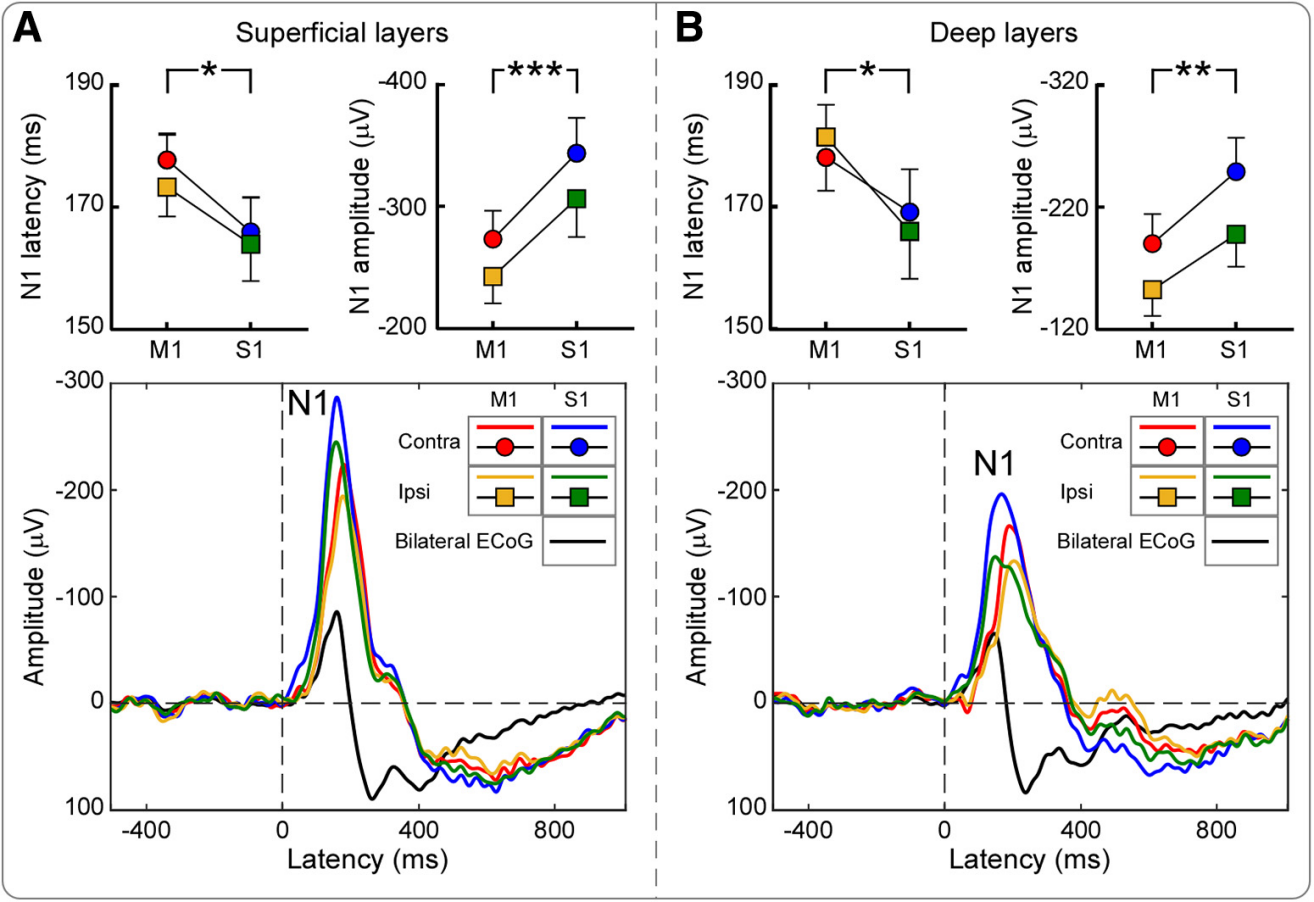
Group-level laser-evoked field potentials in the time domain. Data were simultaneously recorded intracortically and epidurally. ***A***, ***B***, Intracortical data were recorded at superficial (***A***) and deep (***B***) layers from the bilateral S1 and M1 (colored waveforms). Epidural data (signal averaged across two electrodes; black waveform) were recorded from two electrodes placed in between the S1 and M1. For all recording sites, the largest LFP response was a negative wave peaking at ∼170 ms (i.e., N1 wave). N1 amplitude was overall larger in S1 than in M1, as well as larger in the hemisphere contralateral than ipsilateral to laser stimulation. N1 latency was shorter in S1 than in M1, in both hemispheres. *p < 0.05, **p < 0.01, ***p < 0.001. Error bars represent SEM.

In both intracortical and epidural electrodes, laser stimuli evoked a clear negative wave in the time domain (N1 wave; Fig. 2). Latency and amplitude of the N1 wave measured from intracortical electrodes were compared across the four recording sites using a two-way repeated-measures ANOVA, with two within-subject factors (hemisphere, contralateral and ipsilateral to laser stimuli; brain region, S1 and M1). As summarized in Tables 1 and 2, N1 amplitudes measured from superficial layers were strongly modulated by both factors (hemisphere: *F*_(1,15)_ = 11.70, *p* = 0.004, partial η^2^ = 0.455; brain region: *F*_(1,15)_ = 19.8, *p* = 0.001, partial η^2^ = 0.586): N1 amplitude was larger when measured from the S1 and M1 contralateral to the stimulus rather than ipsilateral to the stimulus, as well as larger when measured from S1 than from M1. No hemisphere × brain region interaction was observed. Consequently, N1 amplitude was largest in the contralateral S1 and smallest in the ipsilateral M1. In addition, we found weak evidence that the N1 latency was modulated by the factor brain region (*F*_(1,15)_ = 5.52, *p* = 0.034, Partial η^2^ = 0.283), suggesting that it was shorter in S1 than in M1. Similar results were obtained for N1 amplitudes and latencies when responses were collected at deep layers (Tables 1, 2).

**Table 1.** Laser-evoked LFP responses measured from the superficial and deep layers of the S1 and M1 contralateral and ipsilateral to the stimulated forepaw

	Contralateral M1	Contralateral S1	Ipsilateral M1	Ipsilateral S1
Superficial layers				
N1 amplitude (μV)	−273 ± 23	−343 ± 29	−242 ± 22	−306 ± 31
N1 latency (ms)	177 ± 4	165 ± 5	173 ± 5	164 ± 6
GBO magnitude (100%)	1.44 ± 0.25	1.92 ± 0.28	1.57 ± 0.22	1.56 ± 0.22
Deep layers				
N1 amplitude (μV)	−190 ± 24	−249 ± 28	−153 ± 21	−198 ± 27
N1 latency (ms)	178 ± 5	169 ± 7	181 ± 5	166 ± 8
GBO magnitude (100%)	1.53 ± 0.12	1.70 ± 0.24	1.69 ± 0.20	1.72 ± 0.17

Data are expressed as the mean ±SEM.

**Table 2.** Two-way repeated-measures ANOVA to assess the effect of recording site on laser-evoked LFP responses (2 × 2 ANOVA, with hemisphere (contralateral, ipsilateral) and brain region (S1, M1) as experimental factors)

	Main effects						Hemisphere × brain region interaction		
	Hemisphere			Brain region					
	*F* value	*p* value	Partial η^2^	F value	*p* value	Partial η^2^	*F* value	*p* value	Partial η^2^
Superficial layers									
N1 amplitude	11.70	**0.004**	0.455	19.84	**0.001**	0.586	0.36	0.556	0.025
N1 latency	3.52	0.082	0.201	5.52	**0.034**	0.283	0.50	0.490	0.035
GBO magnitude	0.93	0.350	0.058	7.31	**0.016**	0.328	5.17	**0.038**	0.256
Deep layers									
N1 amplitude	44.15	**<0.001**	0.773	9.47	**0.009**	0.422	2.77	0.120	0.176
N1 latency	0.19	0.667	0.014	4.76	**0.047**	0.254	0.71	0.415	0.048
GBO magnitude	0.83	0.378	0.056	1.05	0.324	0.070	0.17	0.686	0.012

*p* values <0.05 are highlighted in bold.

### Laser-induced oscillations in the gamma band (GBOs)

**Figure 3.**
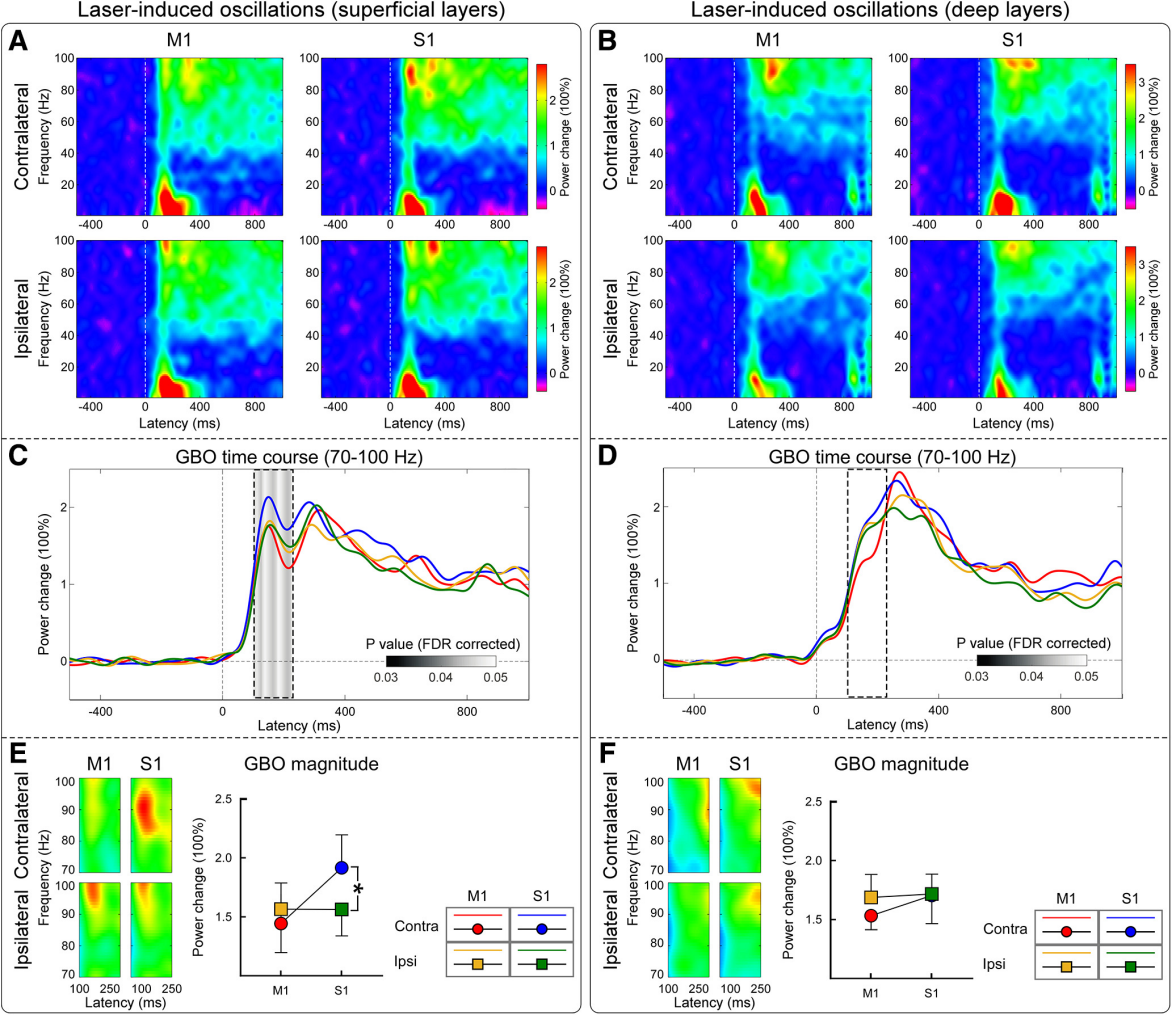
***A–F***, Group-level time–frequency distributions of laser-induced LFP oscillations recorded from superficial (***A***, ***C***, ***E***) and deep (***B***, ***D***, ***F***) layers of the bilateral S1 and M1. ***A***, ***B***, Broadband time–frequency responses recorded from the S1 and M1 (columns) contralateral and ipsilateral to the stimulation side (rows). ***C***, ***D***, Time courses of the power of GBOs were obtained by averaging time–frequency responses elicited by laser stimulation at 70–100 Hz. Gray-shaded areas indicate *p* values (*p* < 0.05, FDR corrected) of the hemisphere × brain region interaction at each time point. Note that only GBOs measured from superficial cortical layers show a clear interaction in the early part of the response (100–220 ms). ***E***, ***F***, GBOs measured in superficial layers (100–220 ms) were larger in contralateral S1 than in contralateral M1, while similar in ipsilateral S1 and bilateral M1. No differences were observed when assessing the magnitude of GBOs measured at deep cortical layers. *p < 0.05. Error bars represent SEM.

Nociceptive stimuli induced a clear enhancement of GBOs in both superficial and deep cortical layers, at all four recording sites (Fig. 3). Time courses of GBO magnitudes showed two distinct poststimulus peaks, especially in superficial layers: an early peak at ∼170 ms; and a late peak at ∼300 ms. We observed moderate evidence that in the time interval 100–220 ms poststimulus the magnitude of GBOs measured from superficial layers was stronger in S1 than in M1, and particularly so in the hemisphere contralateral to the stimulated forepaw (main effect of brain region: *F*_(1,15)_ = 7.31, *p* = 0.016, partial η^2^ = 0.328; hemisphere × brain region interaction: *F*_(1,15)_ = 5.17, *p* = 0.038, partial η^2^ = 0.256). There was no evidence for any effect of recording site on GBOs sampled from deep cortical layers (Tables 1, 2).

### Cross-correlation of GBO between LFP and ECoG

**Figure 4.**
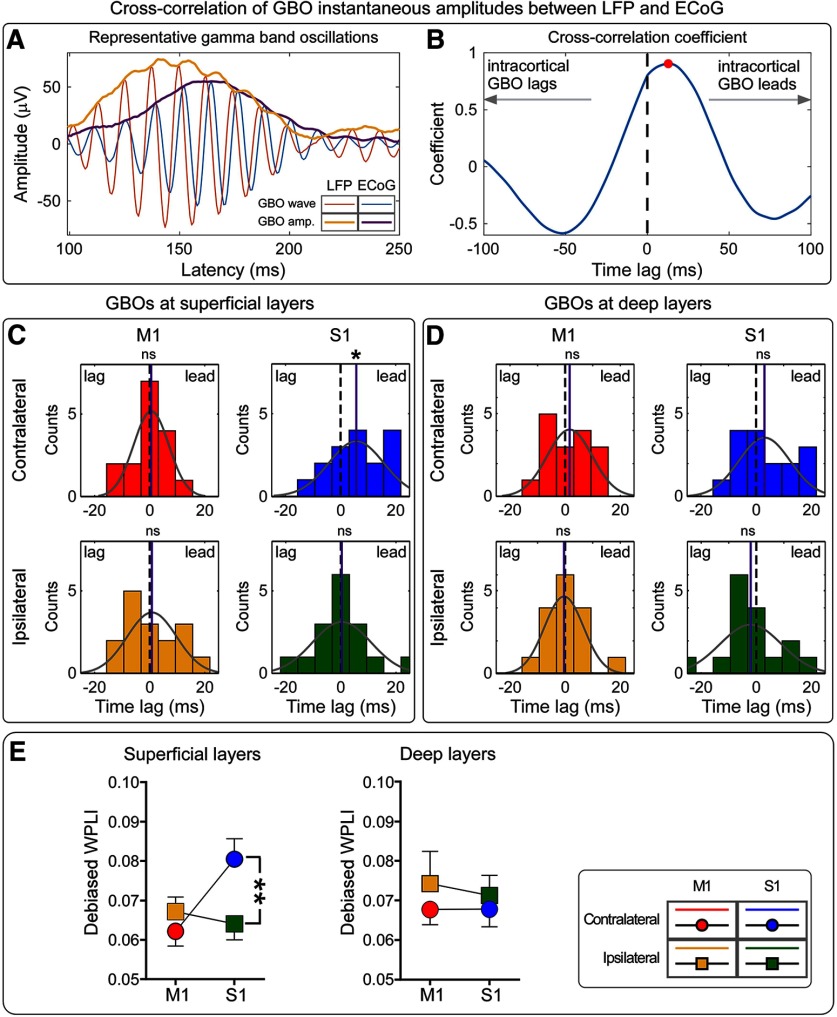
The temporal sequence and phase relationship between GBOs measured intracortically and epidurally. ***A***, Representative GBOs (time, 100–250 ms; frequency, 70–100 Hz) recorded intracortically and epidurally are displayed in red and blue, respectively; their instantaneous amplitudes are displayed in orange and purple, respectively. ***B***, Cross-correlation coefficients of the instantaneous amplitudes of representative intracortical and epidural GBOs. The maximal coefficient occurs at a time lag larger than zero (i.e., red dot), indicating that the instantaneous amplitude of intracortical GBOs leads that of epidural GBOs. ***C***, ***D***, Distribution of time lags at which the cross-correlation coefficients between intracortical and epidural GBOs is maximal. Data are recorded from S1 and M1, contralateral and ipsilateral to stimulation side (***C***, superficial layers; ***D***, deep layers). For each plot, the gray curve represents the normal distribution fitting; the mean of each fitting is marked with a purple line. Wilcoxon rank sum test statistics indicated that only the instantaneous amplitude of GBOs measured from the superficial layers of the contralateral S1 lead that of epidural GBOs. Statistical results are summarized in Extended Data Figure 4-1. ***E***, The debiased WPLI measures the phase relationship between intracortical and epidural GBOs. There was strong evidence that WPLI values calculated for GBOs measured at superficial cortical layers were modulated by an hemisphere × brain region interaction: they were larger in contralateral than in ipsilateral S1, but were similar in contralateral and ipsilateral M1. In contrast, there was no evidence for any effect of recording site on WPLI values at deep cortical layers. Statistical results are summarized in Extended Data Figure 4-2. *p < 0.05, **p < 0.01. ns, Not significant. Error bars represent SEM.

To understand the time relationship between GBOs sampled intracortically and epidurally, we cross correlated their instantaneous amplitude (i.e., their envelope; Fig. 4*A–D*). At superficial layers, the instantaneous amplitude of GBOs measured in the contralateral S1 significantly lead that of the GBOs measured epidurally. Specifically, GBOs in contralateral S1 preceded epidural GBOs by 6.2 ± 2.3 ms (*p* = 0.03, *z* = 2.22, Wilcoxon rank sum test). Importantly, there was no clear evidence of a similar precedence when considering the GBOs measured at any other intracortical location (i.e., from bilateral M1, ipsilateral S1, or deep layers of contralateral S1; all *p* > 0.05, *z* <1.4; Extended Data Fig. 4-1). These results suggest that laser-induced GBOs recorded epidurally (which, notably, show the same functional properties with those recorded using scalp EEG in humans (Hu and Iannetti, 2019)) are preceded by the responses recorded from superficial layers of contralateral S1.

10.1523/JNEUROSCI.0255-20.2020.f4-1 Figure 4-1 Temporal sequence of instantaneous amplitude between laser-induced GBOs measured intracortically and epidurally. Download Figure 4-1, DOCX file

10.1523/JNEUROSCI.0255-20.2020.f4-2 Figure 4-2 Two-way repeated-measures ANOVA to assess the effect of recording site on WPLI values [2 × 2 ANOVA, with hemisphere (contralateral, ipsilateral) and brain region (S1, M1) as experimental factors]. Download Figure 4-2, DOCX file

### Phase relationship of GBO between LFP and ECoG

To explore the phase relationship between GBOs sampled intracortically and epidurally, we estimated their phase consistency using the debiased WPLI (Fig. 4*E*). In superficial cortical layers, we found strong evidence that WPLI values were modulated by a hemisphere × brain region interaction (*F*_(1,15)_ = 12.12, *p* = 0.003, partial η^2^ = 0.447; Extended Data Fig. 4-2). *Post hoc* paired-sample *t* tests showed strong evidence that WPLI values were larger in contralateral than in ipsilateral S1 (*p* = 0.002, Bonferroni corrected, like all other *post hoc t* tests), but similar in contralateral and ipsilateral M1 (corrected *p* = 0.967). In contrast, there was no evidence for any effect of recording site on WPLI values at deep cortical layers (Extended Data Fig. 4-2). These results indicate that GBOs recorded epidurally have a stronger phase consistency with GBOs recorded from superficial layers of contralateral S1 than any other brain regions.

### Laser-evoked spikes in single units

A total of 578 units with clear spike responses were identified in bilateral S1 and M1. Of these 578 units, 227 were putative interneurons (97 in M1 and 130 in S1), and 351 were putative pyramidal neurons (178 in M1 and 173 in S1).

**Figure 5.**
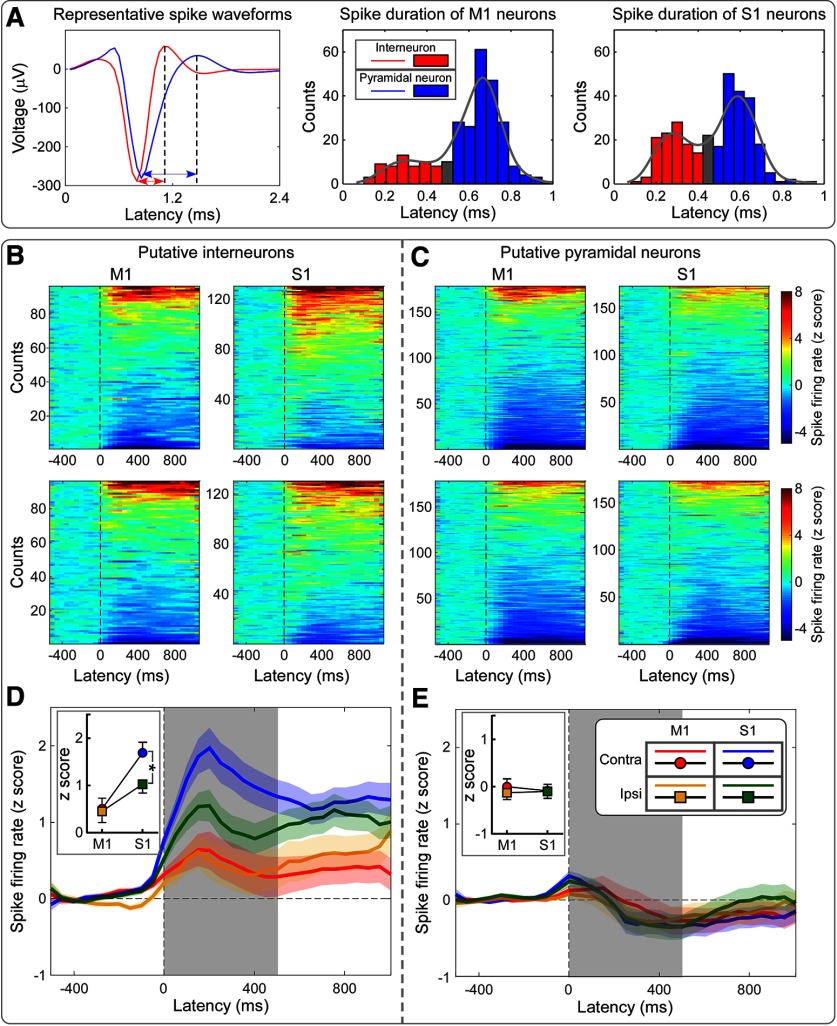
Laser-evoked spikes of putative interneurons and pyramidal neurons at the four recording sites (bilateral S1 and M1). ***A***, Representative spike waveforms of a putative interneuron and a putative pyramidal neuron are displayed in red and blue, respectively (left). Spike durations are marked using double-headed arrows. Note that the spike duration of the putative interneuron is shorter than that of the putative pyramidal neuron. Distributions of spike durations: M1, middle; S1, right. In both regions, spike durations showed a bimodal distribution, which was used to identify cells as putative interneurons (red), pyramidal neurons (blue), and unclassified neurons (gray). ***B***, ***C***, Spike-firing rates of putative interneurons (***B***) and pyramidal neurons (***C***) at the four recording sites. Spike-firing rates, expressed as *z*-scores, were normalized with respect to the baseline (i.e., 500 ms preceding the nociceptive stimulation). Units are sorted along the *y*-axis of each bidimensional plot according to the direction of modulation, from stimulus-induced decrease (bottom) to increase (top) of the firing rate. ***D***, ***E***, Mean firing rates (spike density functions) across all putative interneurons (***D***) and pyramidal neurons (***E***) at each of the four recording sites. In the first 500 ms following nociceptive stimulation, mean firing rates were larger in S1 than in M1, and were larger in contralateral S1 than in ipsilateral S1. In contrast, firing rates of pyramidal neurons were not different. Statistical results are summarized in Extended Data Figure 5-1. *p < 0.05. Error bars represent SEM.

As summarized in Figure 5 and Extended Data Fig. 5-1, nociceptive laser stimuli induced a modulation of the spike-firing rate of putative interneurons. The magnitude of spike-firing rates in the 0–500 ms poststimulus time window was modulated by both hemisphere (with overall higher firing rates in the contralateral hemisphere: *F*_(1, 226)_ = 9.28, *p* = 0.003, partial η^2^ = 0.040) and brain region (with overall higher firing rates in S1 than in M1: *F*_(1,226)_ = 9.47, *p* = 0.002, partial η^2^ = 0.040). Crucially, we observed moderate evidence of a hemisphere × brain region interaction (*F*_(1,226)_ = 6.56, *p* = 0.011, partial η^2^ = 0.028): indeed, *post hoc* paired-sample *t* tests showed strong evidence that firing rates of interneurons were higher in contralateral S1 than in ipsilateral S1 (corrected *p* < 0.001), but were not different in contralateral M1 and ipsilateral M1 (corrected *p* = 0.126). We found no evidence for any effect on firing rate of putative pyramidal neurons (Fig. 5, Extended Data Fig. 5-1).

10.1523/JNEUROSCI.0255-20.2020.f5-1 Figure 5-1 Two-way repeated-measures ANOVA to assess the effect of recording site on normalized spike-firing rates [2 × 2 ANOVA, with hemisphere (contralateral, ipsilateral) and brain region (S1, M1) as experimental factors]. Download Figure 5-1, DOCX file

**Figure 6.**
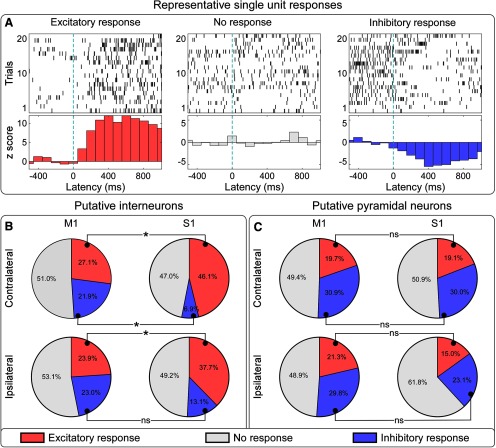
Proportions of neurons showing different types of spiking responses to laser stimulation at the four recording sites. ***A***, Representative neurons showing excitatory (left), lack of (middle), and inhibitory (right) spike responses to laser stimulation. Raw trains of single-trial spike responses are shown in the top plots, and their across-trial averages are shown in the bottom plots, where spike-firing rates are displayed as *z*-scores, binned in 100 ms windows, and normalized to the baseline (−500 to 0 ms relative to the laser stimulation). ***B***, ***C***, Percentages of neurons showing different types of spike responses to nociceptive stimulation, at each of the four recording sites (***B***, interneurons; ***C***, pyramidal neurons). Excitatory, lack of, and inhibitory responses are coded in red, gray, and blue, respectively. The proportion of putative interneurons showing an excitatory response was larger in S1 than in M1, in both hemispheres. The proportion of interneurons showing an inhibitory response was smaller in contralateral S1 than in contralateral M1, but it was similar in the ipsilateral S1 and M1. In contrast, the proportion of pyramidal neurons with different types of responses was not different across the four recording sites. *p < 0.001. ns, Not significant.

By comparing spike-firing rates between prestimulus and poststimulus, we identified the following three types of neuronal responses: increase, lack of modulation, and decrease of firing rate (Fig. 6). We observed strong evidence that there were more putative interneurons showing excitatory responses in contralateral S1 than in contralateral M1 (46.1% vs 27.1%, *p* < 0.001), and in ipsilateral S1 than in ipsilateral M1 (37.7% vs 23.9%, *p* < 0.001). In addition, we observed strong evidence that interneurons with inhibitory responses were less in contralateral S1 than in contralateral M1 (6.9% vs 21.9%, *p* < 0.001), and no evidence that they were less in ipsilateral S1 than in ipsilateral M1 (13.1% vs 23.0%, *p* = 0.074). When considering putative pyramidal neurons, there was no consistent difference in the proportion of units showing excitatory or inhibitory responses between contralateral S1 and M1, as well as between ipsilateral S1 and M1 (all *p* > 0.05; Fig. 6). Overall, these results clearly indicate that putative interneurons had more excitatory and fewer inhibitory responses to laser stimuli in bilateral S1 than in bilateral M1. This was not the case for putative pyramidal neurons.

### Spike-field coherence between spikes and epidural GBOs

**Figure 7.**
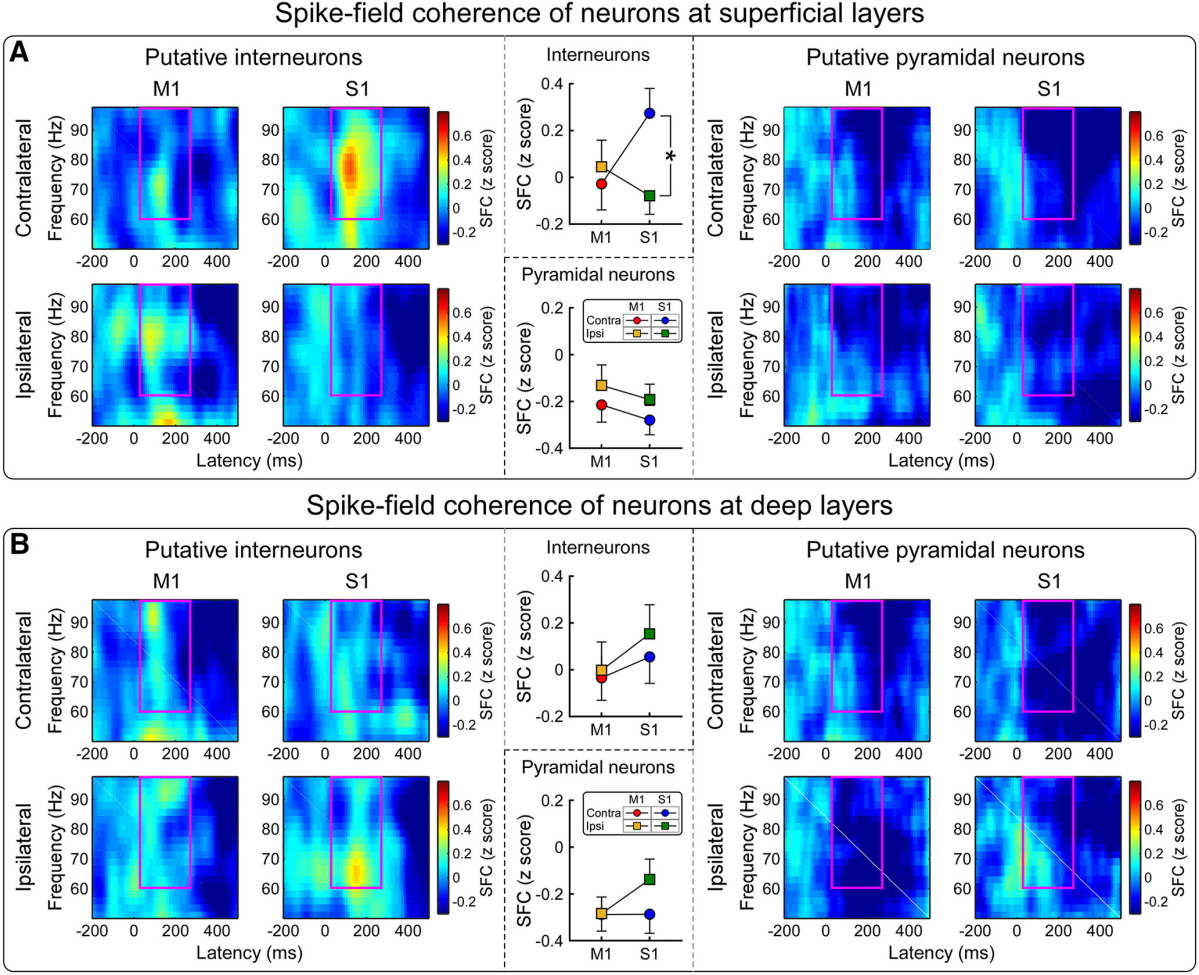
Spike-field coherence (SFC) to test whether laser-induced increases of spike firing in bilateral S1 and M1 occurred at specific phases of the laser-induced GBOs simultaneously recorded from the brain surface. ***A***, ***B***, SFCs of putative interneurons (left) and pyramidal neurons (right) measured at superficial (***A***) and deep (***B***) layers. SFCs, represented as *z*-scores, are normalized to the baseline (i.e., 500 ms preceding the nociceptive stimulation). There was a high coherence between the spike-firing rates of interneurons in the superficial layers of contralateral S1 and the phase of epidural GBOs (60–100 Hz and 100–250 ms, marked using purple rectangles). SFCs of superficial interneurons were larger in contralateral S1 than in ipsilateral S1, but were similar in contralateral and ipsilateral M1. In contrast, SFCs of interneurons at deep layers or of pyramidal neurons at both superficial and deep layers were similar. Statistical results are summarized in Extended Data Figure 7-1. *p < 0.05. Error bars represent SEM.

Spike firing in single units can generate oscillations with a broadband frequency and a power distribution depending on the composition of the active cell types (Buzsáki et al., 2012). For this reason, we tested whether laser-induced GBOs recorded from the brain surface using epidural electrodes (ECoG) were associated with laser-induced increases of spike firing in S1 and M1. To explore this relationship, we calculated the SFC, a measure of how neurons tend to fire spikes at particular phases of GBOs, for each of the four intracortical recording sites. We observed strong evidence for a high coherence between spike-firing rates of interneurons in the superficial layers of S1 and the phase of GBOs measured epidurally, in the time–frequency window of 60–100 Hz and 100–250 ms (Fig. 7). As summarized in Extended Data Fig. 7-1, SFC (expressed as *z*-score) of putative interneurons in superficial layers was modulated by the hemisphere × brain region interaction (*F*_(1,107)_ = 5.97, *p* = 0.016, partial η^2^ = 0.053). *Post hoc* paired-sample *t* tests showed strong evidence that the SFC of putative interneurons in superficial layers was larger in contralateral S1 than in ipsilateral S1 (corrected *p* = 0.004), but not larger in contralateral M1 than in ipsilateral M1 (corrected *p* > 0.999). In contrast, there was no evidence for any effect of recording site on the SFC of putative pyramidal neurons at superficial layers or of both putative interneurons and pyramidal neurons at deep layers (Fig. 7, Extended Data Fig. 7-1). These results provide strong evidence that the spiking activity of interneurons in the superficial layers of the contralateral S1 is the main determinant of laser-evoked GBOs measured epidurally (Fig. 7), which show the same functional properties of GBOs measured on the scalp in human EEG recordings (Gross et al., 2007; Zhang et al., 2012).

10.1523/JNEUROSCI.0255-20.2020.f7-1 Figure 7-1 Two-way repeated-measures ANOVA to assess the effect of recording site on the normalized spike-field coherence [2 × 2 ANOVA, with hemisphere (contralateral, ipsilateral) and brain region (S1, M1) as experimental factors]. Download Figure 7-1, DOCX file

## Discussion

Converging evidence from different research groups indicates that GBOs measured in scalp EEG are one of the most selective markers of perceived intensity of both stimulus-evoked and spontaneous pain (Gross et al., 2007; Zhang et al., 2012; Hu and Iannetti, 2019). However, the low spatial resolution of the neural activity sampled from the brain surface (Pesaran et al., 2018), together with the intrinsic inaccuracy of the EEG source analysis (Nunez and Silberstein, 2000; Lanfer et al., 2012), make it difficult to unequivocally identify the neural origin of GBOs. Specifically, whether GBOs induced by transient nociceptive stimuli are generated from encoding stimulus features in S1 or result from motor-related activity for nocifensive behaviors in M1 is debated (Schulz et al., 2012; Zhang et al., 2012). To address this issue, we simultaneously recorded neural activity both epidurally and intracortically from bilateral S1 and M1 (Fig. 1) in freely moving rats receiving selective stimulation of peripheral nociceptive afferents using radiant heat. We provide four lines of evidence that GBOs induced by nociceptive stimulation are mainly determined by the activity of interneurons located in the superficial layers of S1 contralateral to the stimulated paw.

First, we observed that the magnitude of GBOs was maximal in the superficial layers of S1 contralateral to the stimulatedpaw (Fig. 3*E*). Second, the instantaneous amplitude of GBOs measured from the superficial layers in contralateral S1 selectively preceded the GBOs recorded epidurally (Fig. 4*C*). Third, GBOs recorded epidurally were also more phase consistent with the GBOs recorded from the superficial layers of contralateral S1 than from any other brain regions (Fig. 4*E*). Fourth, only spiking of putative interneurons in the superficial layers of contralateral S1 was coherent with epidural GBOs (Fig. 7*A*). Importantly, no similar relationship with epidural GBOs was observed when examining either intracortical GBOs or spikes measured at the deep layers of primary sensorimotor cortices. This set of results provides the first direct demonstration that GBOs induced by acute somatic noxious stimuli measured at population level reflect neural activity coupled with the spike firing of interneurons located in the superficial layers of the primary somatosensory cortex contralateral to the side of nociceptive stimulation.

### Nociceptive-induced GBOs reflect neural activity in superficial S1 layers

Our results provide the first exploration of the contribution of supragranular versus infragranular cortical layers in the generation of GBOs induced by nociceptive stimulation. The rationale for investigating superficial versus deep cortical layers is the demonstration that GBOs originating from different cortical laminae of primary sensory cortices have been shown to subserve different cognitive states and functions (Adesnik and Scanziani, 2010). GBOs in superficial layers of the S1 contralateral to the stimulated paw displayed not only the largest magnitude, but also the strongest relationship, both in temporal sequence and phase consistency, with the GBOs recorded epidurally (Fig. 4). The selectivity of these effects for superficial layers suggests that S1 neurons, on top of a basic columnar organization the cortex (Mountcastle, 1997), can have different functional roles across different layers. This notion is also supported by the evidence that superficial and deep layers contain somewhat independent networks with distinct functional roles due to laminar-specific connectivity (DeNardo et al., 2015; Ayaz et al., 2019). However, it should be noted that neural activity in superficial layers has strong effects on pyramidal neurons in layer V. For instance, the apical dendrites of pyramidal neurons of the rat neocortex have a spatially restricted low-threshold zone at the level of layers II/III, and the slow dendritic potentials initiated in this zone propagate toward the soma in deep layers, an observation suggesting a critical mechanism for integrating and amplifying sensory and modulatory inputs (Larkum and Zhu, 2002). In general, the layer-specific cortical connectivity of primary sensory?A3B2 twb .33w?> cortices appears to be preserved across different sensory modalities (Ainsworth et al., 2016; Welle and Contreras, 2016; Ayaz et al., 2019; Shiramatsu et al., 2019). For example, sensory input to the primary visual cortex increases GBO magnitude in superficial layers (i.e., supragranular and granular, L2–L4), and the modulation of GBOs in superficial layers does not result in a corresponding change of GBOs in deep layers (Welle and Contreras, 2016). A similar, layer-dependent functional heterogeneity has also been observed in the mouse barrel cortex, with neurons in superficial layers (L2/3) playing a primary role in the multimodal integration of sensory inputs (Ayaz et al., 2019). That the differences in the function of superficial and deep layers are similar across primary sensory cortices of different modalities is likely consequent to the fact that these cortices share striking commonalities in their constituent cell types, synaptic connections, and circuits (Harris and Mrsic-Flogel, 2013). Therefore, it is plausible that GBOs measured at the superficial layers of the contralateral S1 are important for coding noxious stimuli (as the superficial layers of the visual cortex code sensory inputs; Welle and Contreras, 2016) and for integrating information across the different cortical structures generating a pain experience (as the superficial layers of the barrel cortex generate the tactile experience; Ayaz et al., 2019).

### Nociceptive-induced GBOs reflect spiking of S1 interneurons

We observed that laser-induced modulation of spike-firing rates of interneurons, assessed by calculating spike density functions across all recorded units, was maximal in the contralateral S1 (Fig. 5*D*). This modulation was contributed by both units showing an excitatory response and units showing an inhibitory response (Fig. 6*B*). Importantly, we explored whether the respective contribution of interneurons with an excitatory or an inhibitory response was similar across the four recorded cortical regions. We observed that interneurons in the contralateral S1 had the highest percentage of units with an excitatory response and the lowest percentage of units with an inhibitory response (Fig. 6).

To further unravel the relation between GBOs recorded epidurally and the underlying spiking activity, we calculated SFC, a measure of how neurons tend to fire spikes at particular phases of GBOs: high SFC would indicate that spikes and GBOs are temporally structured, and that GBOs are generated by a local spiking source (Pesaran et al., 2018). We observed that only spikes recorded from putative interneurons in the superficial layers of contralateral S1 displayed a strong SFC with epidural GBOs (Fig. 7). This finding provides additional evidence of the primacy of superficial S1 layers contralateral to the stimulated side in determining the GBOs measured epidurally. Also, it shows that putative interneurons, but not putative pyramidal neurons, are important for GBO generation. Our observation that epidural GBOs largely reflect S1 interneurons fits well with the general notion that fast-spiking, parvalbumin-expressing, soma-inhibiting interneurons play a key role in the generation of GBOs in other sensory modalities (Bartos et al., 2007; Wang, 2010; Buzsáki et al., 2012). In addition, and specifically for nociception, when optogenetics is used to induce rhythmic firing of parvalbumin-expressing interneurons in S1, the power of GBOs in LFP recordings is enhanced (Tan et al., 2019). It is fascinating that such GBO enhancement results in an increase in nociceptive sensitivity in freely moving mice (Tan et al., 2019). These results, together with our current findings, suggest that previous descriptions of correlation between GBO magnitude and the intensity of both pain reports in humans and pain-related behaviors in rodents (Peng et al., 2018; Hu and Iannetti, 2019) are subserved by the activation of S1 interneurons.

### Advantages, limitations, and future directions

By simultaneously recording epidural and intracortical neural activity in freely moving rats, we developed an effective model to assess the relationship of neural responses at different spatial scales. Specifically, the possibility of recording nociceptive-induced GBOs at the mesoscopic scale of LFPs allows bridging of the gap between the macroscopic scale of EEG/ECoG and the microscopic scale of single-unit spiking. We provide the first demonstration that scalp GBOs, one of the most promising biomarkers of pain derived by a measure of neural activity at population level, largely reflect the activity of interneurons in the superficial layers of the S1 contralateral to where a nociceptive stimulus is applied. Combining this electrophysiological model with optogenetics will allow determination of the contribution of specific interneuron subtypes and networks to the GBO generation (Chen et al., 2017). In addition, the development of approaches to modulate pain-related GBOs in superficial cortical layers [e.g., via transcranial alternating current stimulation and transcranial magnetic stimulation (TMS)], coupled with neurofeedback (Misselhorn et al., 2019; Cabral-Calderin and Wilke, 2020), could provide a promising avenue for effective pain treatment. Indeed, some of these neuromodulations (e.g., TMS) suppress dendritic activity in superficial cortical layers, by activating dendrite-targeting inhibitory GABAergic interneurons (Murphy et al., 2016).

The current study has at least three important caveats. First, we measured intracortical neural activity using tungsten microelectrodes that were progressively moved using a microdrive—an approach that does not allow an optimal control of the depth of the recording tip of the electrode. For this reason, we were only able to broadly discriminate responses in superficial versus deep cortical layers, but were not able to assign responses to individual cortical layers. In future studies, it will be necessary to identify the local generators of GBOs by calculating the current source density of neural activity sampled using silicon probes with layer-specific spatial resolution (Buzsáki et al., 2012; Pesaran et al., 2018). A second caveat is that we limited the exploration of the relationship between epidural and intracortical neural activity only to primary sensorimotor regions, since the motivation of the study was to test the alternative hypotheses that nociceptive-induced GBOs sampled at scalp level are generated in S1 or M1. Future exploration of other cortical regions that respond to transient nociceptive stimulation will allow testing the contribution of structures other than primary sensorimotor cortices to GBOs measured at both epidural and scalp levels. The last caveat is particularly relevant when considering that GBOs whose magnitude tracks the time-varying fluctuations of the intensity of tonic pain in healthy subjects (Schulz et al., 2015) and in patients with chronic pain (Zhou et al., 2018; May et al., 2019) have been described to originate from the prefrontal cortex and cerebellum, and not from the primary sensorimotor cortices (Schulz et al., 2015; Zhou et al., 2018; Ma et al., 2019; May et al., 2019). This discrepancy might dovetail with the difference of pain types between the above-mentioned studies and the current study, which is also reflected in the different patterns of brain activation observed during chronic pain vs. transient cutaneous pain (Mouraux and Iannetti, 2018, their Fig. 4). For this reason, the current findings cannot be generalized as reflecting tonic and chronic pain.
