# Supplementary material for: The Neural Origin of Nociceptive-Induced Gamma-Band Oscillations
Source: J Neurosci. 2020 Apr 22;40(17):3478–90. doi: 10.1523/JNEUROSCI.0255-20.2020 (PMC7178916; doi:10.1523/JNEUROSCI.0255-20.2020)
Supplement: Figure 4-1 [file ns-JN-RM-0255-20-s01.docx]

**Figure 4-1**. Temporal sequence of instantaneous amplitude between laser-induced GBOs measured intracortically and epidurally.

|  | Contralateral M1 | Contralateral S1 | Ipsilateral M1 | Ipsilateral S1 |
| --- | --- | --- | --- | --- |
| *Superficial layers* |  |  |  |  |
| Time lag (ms) | -0.39±1.60 | 6.17±2.29 | 1.14±2.20 | 1.36±2.43 |
| p value | 0.84 | **0.03** | 0.64 | 0.88 |
| z value | -0.21 | 2.22 | 0.47 | 0.15 |
| *Deep layers* |  |  |  |  |
| Time lag (ms) | 2.59±2.05 | 2.58±2.58 | -0.74±1.86 | -3.06±3.00 |
| p value | 0.16 | 0.33 | 0.68 | 0.28 |
| z value | 1.39 | 0.98 | -0.41 | -1.08 |

p value <0.05 is highlighted in bold.
