# Supplementary material for: The Neural Origin of Nociceptive-Induced Gamma-Band Oscillations
Source: J Neurosci. 2020 Apr 22;40(17):3478–90. doi: 10.1523/JNEUROSCI.0255-20.2020 (PMC7178916; doi:10.1523/JNEUROSCI.0255-20.2020)
Supplement: Figure 4-2 [file ns-JN-RM-0255-20-s02.docx]

**Figure 4-2**. Two-way repeated-measures ANOVA to assess the effect of recording site on WPLI values (2×2 ANOVA, with ‘hemisphere’ [contralateral, ipsilateral] and ‘brain region’ [S1, M1] as experimental factors).

|  | Main effects | | | | | | Hemisphere × Brain region  interaction | | |
| --- | --- | --- | --- | --- | --- | --- | --- | --- | --- |
|  | Hemisphere | | | Brain region | | |  |  |  |
|  | F value | p value | Partial η^2^ | F value | p value | Partial η^2^ | F value | p value | Partial η^2^ |
| Superficial layers | 1.822 | 0.197 | 0.108 | 2.638 | 0.125 | 0.150 | 12.122 | **0.003** | 0.447 |
| Deep layers | 0.622 | 0.443 | 0.043 | 0.199 | 0.663 | 0.014 | 0.153 | 0.702 | 0.011 |

p value <0.05 is highlighted in bold.
