# Supplementary material for: The Neural Origin of Nociceptive-Induced Gamma-Band Oscillations
Source: J Neurosci. 2020 Apr 22;40(17):3478–90. doi: 10.1523/JNEUROSCI.0255-20.2020 (PMC7178916; doi:10.1523/JNEUROSCI.0255-20.2020)
Supplement: Figure 5-1 [file ns-JN-RM-0255-20-s03.docx]

**Figure 5-1**. Two-way repeated-measures ANOVA to assess the effect of recording site on normalized spike firing rates (2×2 ANOVA, with ‘hemisphere’ [contralateral, ipsilateral] and ‘brain region’ [S1, M1] as experimental factors).

|  | Main effects | | | | | | Hemisphere × Brain region  interaction | | |
| --- | --- | --- | --- | --- | --- | --- | --- | --- | --- |
|  | Hemisphere | | | Brain region | | |  |  |  |
|  | F value | p value | Partial η^2^ | F value | p value | Partial η^2^ | F value | p value | Partial η^2^ |
| Interneurons | 9.28 | **0.003** | 0.040 | 9.47 | **0.002** | 0.040 | 6.56 | **0.011** | 0.028 |
| Pyramidal neurons | 0.78 | 0.378 | 0.002 | 0.02 | 0.884 | 0.000 | 0.53 | 0.466 | 0.002 |

p values <0.05 are highlighted in bold.
