# Supplementary material for: The Neural Origin of Nociceptive-Induced Gamma-Band Oscillations
Source: J Neurosci. 2020 Apr 22;40(17):3478–90. doi: 10.1523/JNEUROSCI.0255-20.2020 (PMC7178916; doi:10.1523/JNEUROSCI.0255-20.2020)
Supplement: Figure 7-1 [file ns-JN-RM-0255-20-s04.docx]

**Figure 7-1**. Two-way repeated-measures ANOVA to assess the effect of recording site on the normalized spike-field coherence (2×2 ANOVA, with ‘hemisphere’ [contralateral, ipsilateral] and ‘brain region’ [S1, M1] as experimental factors).

|  | Main effects | | | | | | Hemisphere × Brain region  interaction | | |
| --- | --- | --- | --- | --- | --- | --- | --- | --- | --- |
|  | Hemisphere | | | Brain region | | |  |  |  |
|  | F value | p value | Partial η^2^ | F value | p value | Partial η^2^ | F value | p value | Partial η^2^ |
| *Superficial layers* | | | | | | | | | |
| Interneurons | 2.58 | 0.112 | 0.023 | 0.56 | 0.454 | 0.005 | 5.97 | **0.016** | 0.053 |
| Pyramidal neurons | 1.73 | 0.189 | 0.009 | 0.64 | 0.426 | 0.003 | 0.001 | 0.976 | 0.000 |
| *Deep layers* |  |  |  |  |  |  |  |  |  |
| Interneurons | 1.37 | 0.245 | 0.014 | 0.28 | 0.599 | 0.003 | 0.10 | 0.751 | 0.001 |
| Pyramidal neurons | 1.29 | 0.258 | 0.009 | 0.72 | 0.397 | 0.005 | 1.15 | 0.285 | 0.008 |

p value <0.05 is highlighted in bold.
